# Trends in Occupational Infectious Diseases in South Korea and Classification of Industries According to the Risk of Biological Hazards Using K-Means Clustering

**DOI:** 10.3390/ijerph191911922

**Published:** 2022-09-21

**Authors:** Saemi Shin, Won Suck Yoon, Sang-Hoon Byeon

**Affiliations:** 1 Research Institute of Health Sciences, Korea University, Seoul 02841, Korea; 2 Allergy and Immunology Center, Korea University, Seoul 02841, Korea; 3 School of Health and Environmental Science, Korea University, Seoul 02841, Korea

**Keywords:** biological hazard, industrial accident, risk assessment, k-means clustering

## Abstract

Against the backdrop of the COVID-19 pandemic, it is necessary to identify these risks and determine whether the current level of management is appropriate to respond to the risk of biological hazards depending on the occupation. In this study, the incidence and fatality rates of occupational diseases were calculated using industrial accident statistics of South Korea, and trends by year using joinpoint regression and relative risk by industry using k-means clustering were evaluated for infectious diseases. We found that infectious diseases had the third highest incidence and fourth highest fatalities among all occupational diseases. In the incidence rate, joinpoints appeared in 2009 and 2018, and the annual percent change changed to 7.79, −16.63, and 82.11. The fatality rate showed a consistent increase with an annual percent change of 4.37, but it was not significant. Industries were classified into five groups according to risk, and the legal control measures of certain industries were not sufficient. Follow-up studies are needed to rectify the structural limitations of industrial accident statistics.

## 1. Introduction

Against the backdrop of the recent COVID-19 pandemic, interest in biological hazards has increased throughout South Korea. In South Korea, a total of 47,816 articles on COVID-19 were searched in 10 online newspapers and 3 online broadcasters from 31 December 2019 to 11 March 2020 [1], the period from the first reports of COVID-19 to the time of the pandemic declaration, generating a buzz volume of 2.6 million documents and comments from 20 January 2020 to 8 March 2020 [2]. Many studies have attempted to identify occupations with a high risk of COVID-19 outbreak/infection in South Korea [3,4]; international studies have also aimed to identify such occupations and estimate the number of workers at risk of contracting COVID-19 [5,6]. Accordingly, some occupations have a higher risk of COVID-19 infection than others. Depending on the characteristics of each occupation, the degree of exposure to hazards, including biological hazards or risk of disease, may vary.

Similar to the situation in Europe [7], in South Korea, biological hazards are treated as a minor occupational health issue. The targets of national health surveillance systems for occupational health, namely work environment monitoring and special health examinations, are usually limited to physical and chemical hazards. However, as COVID-19 has become a matter of major concern in society given its major impact on jobs, many studies have attempted to determine how different occupations affect the workers’ risk of contracting COVID-19, and the need to actively discuss ways to control biological hazards in the workplace is being realized.

Currently, in accordance with the Occupational Safety and Health Act, the risk of biological hazards is systematically controlled only in certain tasks, workplaces, or work environments (Table 1). However, it is known that COVID-19 can spread in workplaces that are outside the system [3,4]. To effectively respond to the risk of biological hazards, it is necessary to identify the risk of infection posed by each occupation and determine whether the current level of control is appropriate. Studies on biological hazards in different industries or occupations have been conducted using data from national statistics and disease prevention programs in various countries, such as South Korea [8], the United States [9], and the United Kingdom [10]; many studies have reviewed cases of occupational diseases caused by biological hazards [11,12]. However, to our knowledge, no study has attempted to objectively quantify the relative risks or ranking of biological hazards.

However, several problems arise when researchers attempt to quantify the risk posed by biological hazards. First, Burzoni’s review [13] of methods for evaluating biological hazards in the workplace concluded that the risk of biological hazards has no methodological context and that there is no approach yet to incorporate the variables required for a comprehensive evaluation. In addition, the methods collected in Burzoni’s review are methods of precautionary risk assessment; however, in South Korea, there is no health surveillance system for biological hazards, so there is a lack of exposure data systematically collected throughout the entire workplace. Second, limited data are available regarding occupations in South Korea. The data collected according to the Occupational Safety and Health Act do not include the classification of occupations [14]. In South Korea, industrial classification is the basis for collecting and analyzing all legal data related to occupational safety and health.

Therefore, we devised a strategy to address these problems. Food hygiene is an area that involves active assessment of the risk of biological hazards. Incidence and fatality rates have been used as variables in risk assessment in the field of food hygiene, such as by the European Food Safety Authority [15,16]. Evaluating the risk from each industry using the results such as incidence and fatality rates that have already been generated is also an effective strategy. Industrial Accident Statistics (National Approved Statistics No. 38001), a nationally approved database in South Korea, publishes the number of workers, illnesses, and fatalities caused by various hazards, including biological hazards, each year for each industry division [17,18,19]. Using industrial accident statistics, it is possible to calculate the incidence and fatality rates of biological hazards for each industry.

There are no criteria for determining the degree or grade of the risk, incidence rate, or fatality rate in the workplace in South Korea. In the absence of prior information about the absolute levels of risk characterization, workplaces can be grouped according to the similarity of each value in incidence and fatality and the practical characteristics of the groups can be explored to determine the risk values. K-means clustering is a popular unsupervised technique used to identify similarities between objects based on distance vectors; it is suitable for small datasets [20] and has been used in several recent risk assessment studies [21,22,23,24,25]. K-means clustering is applicable only when the number of groups is determined. Although the number of groups is not generally determined when classifying the incidence and fatality rates, several methods have been developed to determine the optimal number of groups [26], such as the elbow method [27].

The incidence and fatality rates of biological hazards obtained from industrial accident statistics can be usefully used in addition to classifying risks by industry. There have been previous studies that studied the risk of biological hazards through industrial accident statistics, and trend analysis that follows the literature can be performed. It can also provide insight into the appropriate management of risks posed by biological hazards for the entire worker population and trends over time.

This study aimed to analyze the scale and yearly trends of infectious diseases by calculating the incidence and fatality rates using classifications of occupational diseases, including infectious diseases, from industrial accident statistics and to discuss appropriate control strategies for each risk group.

## 2. Materials and Methods

Information on the number of workers, illnesses, and fatalities by year and industry and detailed classifications of occupational diseases from 2001 to 2020 were extracted from the published industrial accident statistics in South Korea. For each year, industry, and detailed classification of occupational diseases, the fatality rate was calculated as the ratio of the number of deaths to the number of reported cases of illness, and the incidence rate was calculated as the ratio of the number of illnesses to the number of workers.

We used joinpoint regression [28] to analyze incidence and fatality rate trends. A series of joined straight lines were fitted for the incidence and fatality rates of infectious diseases and all occupational diseases and the ratio of the incidence and fatality rates of infectious diseases to all occupational diseases. We performed a log transformation on the dependent variable and set the maximum number of join points to 3. All processes related to joinpoint regression were performed through a joinpoint regression program (ver. 4.9.1.0, National Cancer Institute, Rockville, MD, United States).

Using k-means clustering, the risk rating of biological hazards for each industry was determined according to the log values of the fatality and incidence rates attributed to the infectious diseases over the entire study period. When zero occurred in log-transformed data, we followed the most common practice of solving by adding a very small constant c, such as half of the smallest nonzero value [29]. Specifically, the value was replaced with 1/2 of the minimum value other than 0, even when the incidence rate was not calculated because the number of workers was 0 or when the fatality rate was not calculated because the number of illnesses was 0. The number of clusters was determined by exploratory rather than formalized methods, such as reviewing several value indices derived using NbClust packages in R program (ver. 4.2.1, R foundation, Indianapolis, IN, Unitited States) [30] and observing real data forms. The task, workplace, and work environment designated for each industry division by the Korea Occupational Safety and Health Act were reviewed to determine whether there were any legal control obligations.

## 3. Results

In all, 91 industries were surveyed. Industrial accident statistics reporting is a separate system from the International Standard Industrial Classification or Standard Industrial Classification localized by each country, where industries are classified according to the industrial accident rate every year as per the Korean Ministry of Employment and Labor’s notice. Twelve industries, including food manufacturing, metal smelting, shipbuilding, and repair were surveyed from 2001 to 2020, and seven industries, including pharmaceuticals, cosmetics, briquettes, and petroleum products, were surveyed for the first time in 2020.

**Figure 1:**
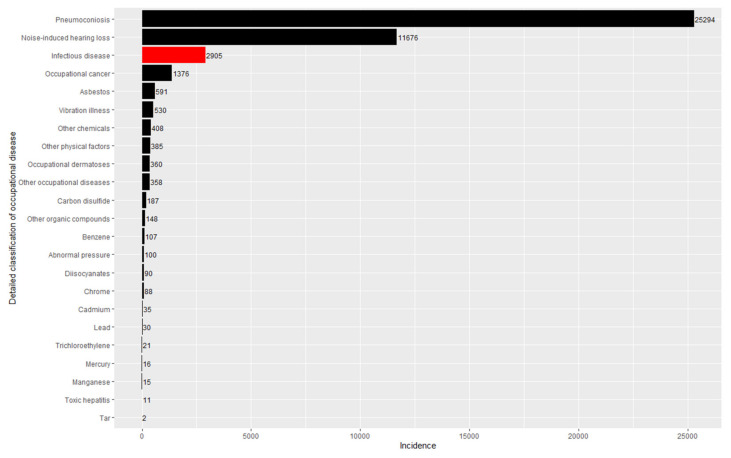
Incidences of occupational diseases (2001–2020).

**Figure 2:**
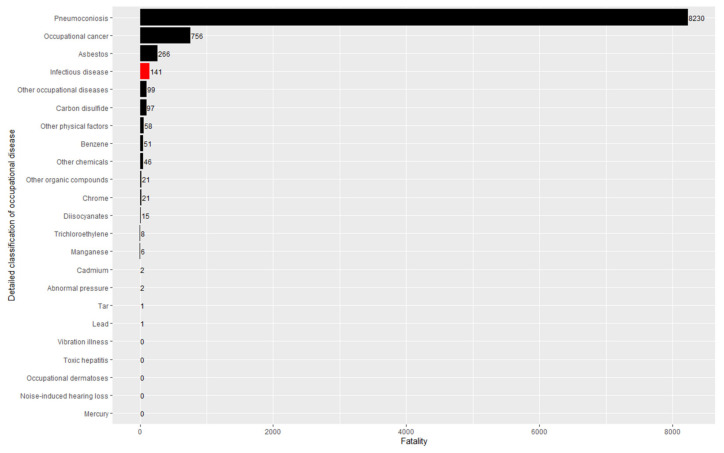
Fatalities of occupational diseases (2001–2020).

Table 2 summarizes the number of illnesses, fatalities, incidence rates, and fatality rates over the entire study period for all 23 classifications of occupational diseases in the industrial accident statistics. Infectious diseases ranked third in terms of incidence with 2905 incidences (6.5%) out of the 44,733 incidences of all occupational diseases (Figure 1) and ranked fourth in terms of fatality with 141 (1.5%) out of 9,521 fatalities (Figure 2). Pneumoconiosis topped the list, with more than half of the incidences (25,294, 56.5%) and fatalities (5230, 54.9%).

Table 3 shows the annual incidences, fatalities, and number of workers as well as the incidence and fatality rates and rates of change from the previous year calculated therefrom for infectious disease among the classifications of occupational diseases targeting all workers. The incidence rate was the highest at 427 (3.08 case per 100,000 employees) in 2009, the fatality was 17 in 2010, and the fatality rate was the highest at 13.1% in 2014.

**Figure 3:**
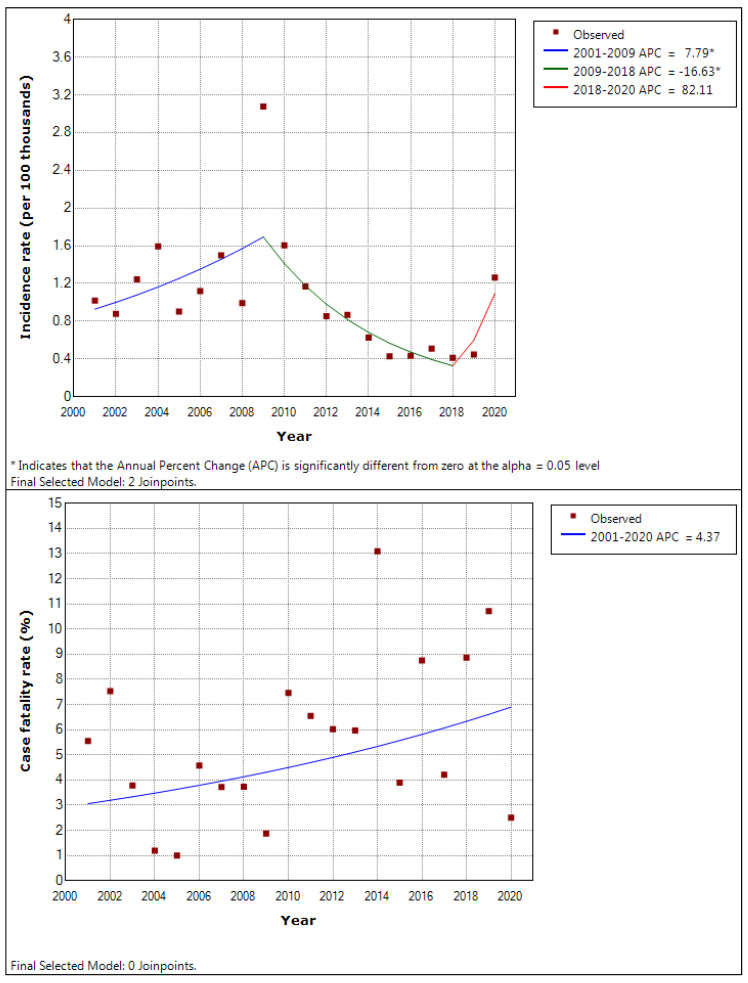
The incidence and fatality rate trends of occupational infectious diseases analysed by joinpoint regression.

The incidence and fatality rate trends of occupational infectious diseases are shown in Figure 3. In the incidence rate, joinpoints appeared in 2009 and 2018, and the annual percent change changed to 7.79 (*p*-value = 0.039), −16.63 (*p*-value < 0.001), and 82.11 (*p*-value = 0.067). The fatality rate showed a consistent increase with an annual percent change of 4.37, but it was not significant (*p*-value = 0.109).

For all occupational disease targeting all workers, Table 4 shows the annual incidences, fatalities, number of workers, and incidence and fatality rates calculated therefrom as well as the ratios of the incidence and fatality rates of infectious diseases to all occupational diseases by year.

**Figure 4:**
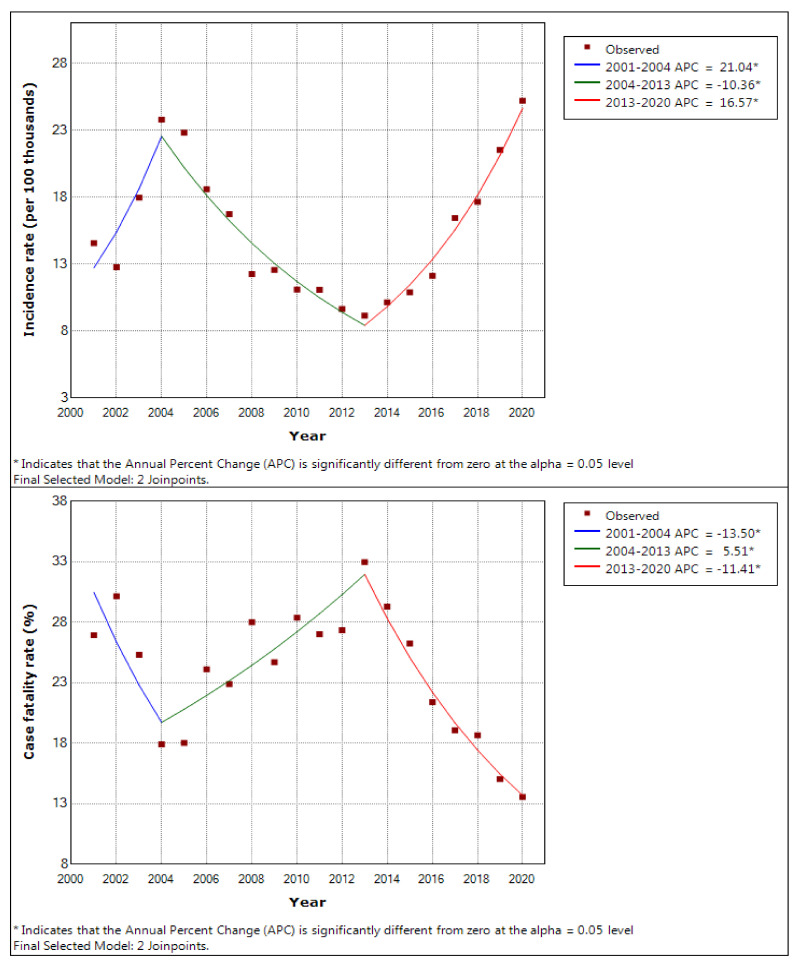
The incidence and fatality rate trends of all occupational diseases analysed by joinpoint regression.

The incidence and fatality rate trends of all occupational diseases are shown in Figure 4. In the incidence rate, joinpoints appeared in 2004 and 2013, and the annual percent change changed to 21.04 (*p*-value = 0.014), −10.36 (*p*-value < 0.001), and 16.57 (*p*-value < 0.001). In the fatality rate, joinpoints also appeared in 2004 and 2013, and the annual percent change changed to −13.50 (*p*-value = 0.033), 5.51 (*p*-value = 0.002), and −11.41 (*p*-value < 0.001).

**Figure 5:**
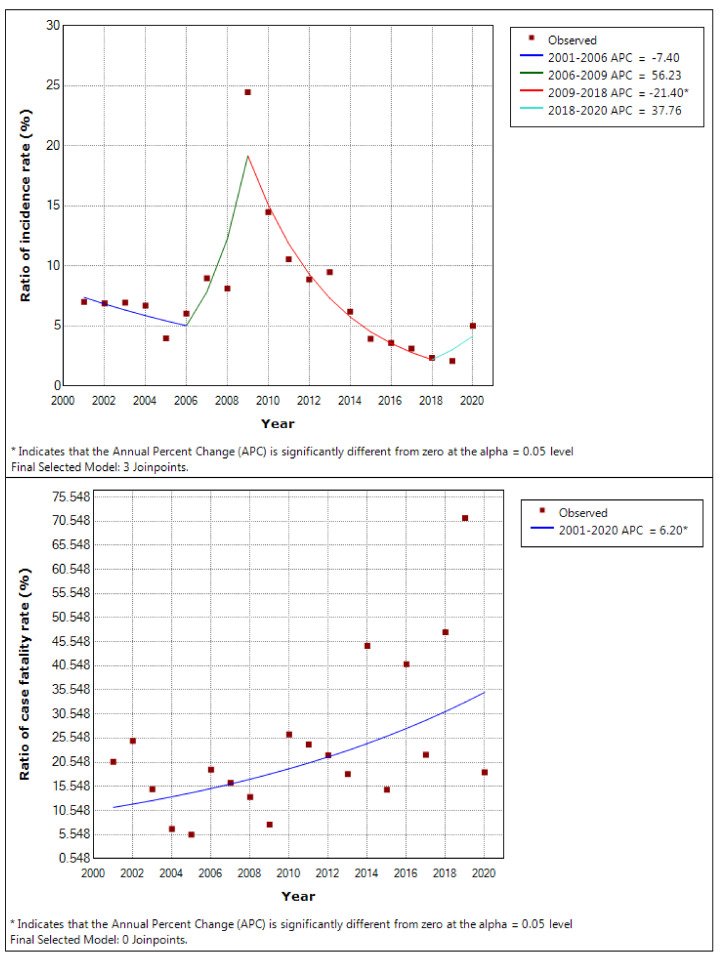
The trends of ratios of the incidence and fatality rate of infectious disease to all occupational diseases analysed by joinpoint regression.

The trends of ratios of the incidence and fatality rate of infectious disease to all occupational diseases are shown in Figure 5. In the incidence rate, joinpoints appeared in 2006, 2009, and 2018, and the annual percent change changed to −7.40 (*p*-value = 0.154), 56.23 (*p*-value = 0.074), −21.40 (*p*-value < 0.001), and 37.76 (*p*-value = 0.181). The fatality rate showed a significantly consistent increase with an annual percent change of 6.20 (*p*-value = 0.012).

**Figure 6:**
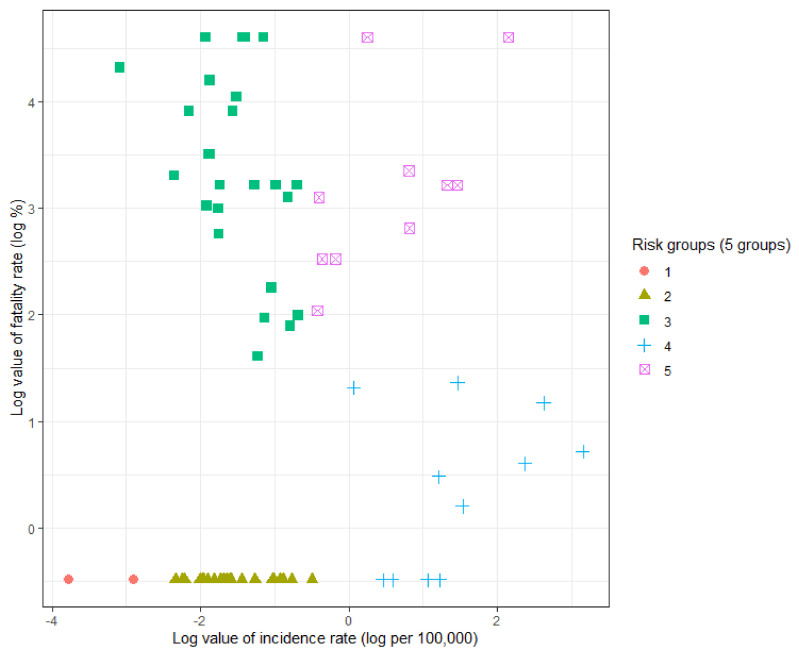
The distribution of incidence and fatality rates and risk groups among industries.

When dividing the risk groups according to the incidence and fatality rates by industry division for the entire study period of infectious diseases, five groups were generated. The optimal value was shown in Pseudo T2 of Duda and Hart [31]. In addition, when five groups were selected, quadrants orthogonal to the axes of incidence and fatality rates can be drawn, and data can be intuitively clustered based on high and low incidence and fatality rates. The characteristics of the five groups (groups 1–5) are as follows: (almost) zero risk, low incidence/low fatality, low incidence/high fatality, high incidence/low fatality, and high incidence/high fatality, respectively (Table 5). The distribution of the incidence, fatality rates, and risk groups among the industries is shown in Figure 6. Industry division by risk group and the incidence and fatality rates by industry division are shown in Table 6.

In Groups 1 and 2, there were no fatalities caused by biological hazards. In Group 1 (30 industries), there were no incidences, except in one industry, and the incidence rate of that division was very low at 5.46 × 10^−2^ cases per 100,000 persons. In Group 2 (22 industries), the incidence rate was low at 4.64 × 10^−1^ cases per 100,000 persons. In Group 3 (25 industries), the incidence rate was low at 4.97 × 10^−1^ cases per 100,000 persons, but it was composed of industries with fatalities, and the fatality rate ranged from 5 to 100%, with an average rate of 40.3%. In Group 4 (11 industries), the incidence rate ranged from 1.80 cases to 23.57 cases per 100,000 persons, and the fatality rate ranged from 0 to 3.92%. The incidence rate of Group 5 (10 industries) ranged from 6.56 × 10^−1^ cases to 8.63 cases per 100,000 persons, and the fatality rate ranged from 7.69% to 100%, with an average fatality rate of 35.0%. In the case of agriculture, forestry, sanitation, and similar service businesses, the incidence rate exceeded 10 per 100,000 persons, and the industry with the highest incidence rate was forestry.

## 4. Discussion

Among the classifications of occupational diseases, the infectious disease classification ranked third with regard to the incidence and incidence rate; it ranked 16^th^ in terms of the fatality rate and fourth in terms of fatality because of the high incidence rate. Even if occupational diseases caused by biological hazards are grouped under a single classification and occupational diseases caused by physical or chemical hazards are divided into multiple classifications, the incidence of infectious diseases is higher than that of occupational diseases caused by physical hazards, except noise-induced hearing loss, chemical hazards, and pneumoconiosis.

Chung [8] analyzed the raw data of industrial accident statistics from 2000 to 2007. The incidence rate decreased from 8.0% to 6.5%, on average, over the entire study period. The increasing trend changed to a decreasing trend after 2009, and then turned to an increasing trend again after 2018 in this study. In 2009 and 2020, the incidence increased sharply, and joinpoints and increasing intervals were induced. Although the industrial accident statistics do not publish the specific causes or diseases of each industrial accident, there were swine flu and COVID-19 pandemics in 2009 and 2020, respectively, and it was confirmed that some jobs, including those of healthcare workers, were significantly affected by the COVID-19 pandemic [32]. There is a high possibility of a surge in the incidence owing to public health issues as it is still a disease with high incidence and fatality as of 2020. The fatality rate was not significant, but there was a consistent increase over the period. It did not decrease even in 2013–2020 when the fatality rate of all occupational diseases decreased. The ratio of the fatality rate of infectious diseases to the fatality rate of occupational diseases shows a significant increase over the entire period. Thus, it is unreasonable to assume that the risk of biological hazards is decreasing and that administrative resource allocation can be reduced.

In the evaluation of risks by industry division, the industries in Groups 1 to 3 usually have not been mentioned in the existing literature as major biohazard-generating industries, and it is difficult to confirm whether a major source of infection exists in the workplace. No fatal results were observed in Groups 1 and 2. However, the problem is that the average fatality rate was the highest in Group 3 throughout the entire study period.

**Figure 7:**
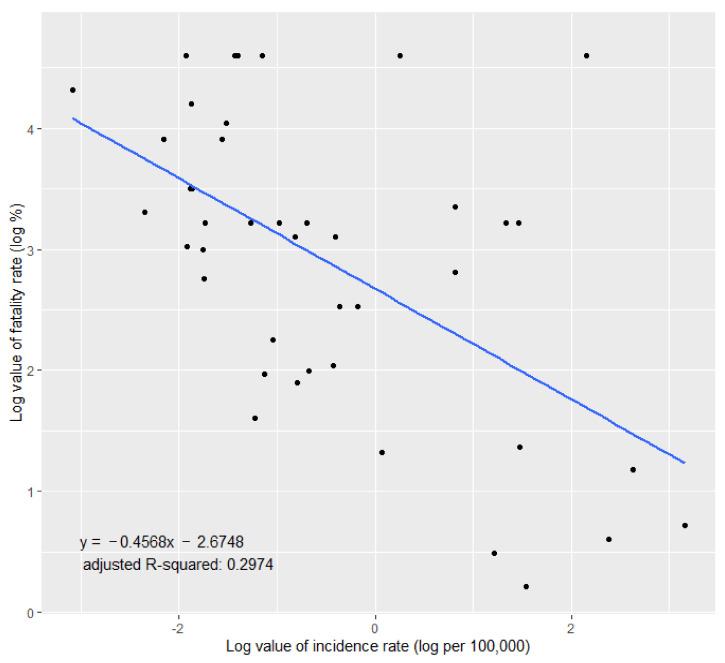
The relationship between the logarithmic value of the incidence rate and fatality rate.

Industrial accident statistics do not directly represent health status but are the result of industrial accident approval, and there is a possibility that bias exists. Figure 7 shows the log values of the fatality and incidence rates of each industry with fatalities. A high negative correlation was observed between the log value of the incidence rate and mortality rate (*p* < 0.001). For industries that were not well known in the past, the incidence of biological hazards may be underestimated due to rare applications or approvals of industrial accidents; however, the severity of the results may increase due to relatively insufficient awareness and response to biological hazards. In order to reduce fatal consequences by preventing outbreaks and responding quickly to incidents, it is necessary to identify and eliminate possible biological hazards in the long term.

Groups 4 and 5, which are groups with high incidence rates, consisted of many industries that are known to involve biological risks, some of which are legally managed. Several case reviews have been published on occupations or industries with biological risks. Corrao [33] and Lim [34] conducted a narrative review of occupational biological risks using data on occupational diseases caused by biological hazards. In Corrao’s review, healthcare, laboratory, dentistry, farming, cattle breeding, waste, wastewater, sewer, and biotechnological industries were considered high-risk industries. Lim considered healthcare, laboratory, agriculture, fishery, forestry, and animal care workers as high-risk workers. Chung [8] considered health and welfare workers, agriculture/forestry worker, other outdoor workers, waste handlers, and overseas dispatchers as high-risk workers according to the Korean industrial accident report. 

Industries designated as high-risk industries were included in both Groups 4 and 5. Given that few industries in these groups are already controlled, there is a need for control policies for all industries within these groups. In Group 4, wood product manufacturing industry; transport-affiliated industry, named as “railroad, air transportation, warehousing, and transportation-related service business”; and sanitation-affiliated industry, named as “comprehensive building management, sanitation and similar service business” are not subject to legal control. In Group 5, sanitation-affiliated industries, named as “comprehensive management of buildings, etc. business”; facility management business and business service; sanitation and similar service business; sawmill and veneering manufacturing; manufacturing of measuring instruments, optical instruments, and other precision instruments; and overseas dispatchers are excluded from legal control. However, overseas dispatchers may be controlled in accordance with quarantine laws.

The biological risks of these industries have been sufficiently identified in previous studies. It is known that industries dealing with wood, such as the sawmill industry, involve the risk of exposure to microorganisms in wood [35,36,37,38,39,40], and the waste is known to be used by microorganisms that breed in decayed organic matter [41,42,43]. The division name of “precision instrument manufacturing” is somewhat broad, but it includes medical device manufacturing, and medical devices that utilize biomaterials are likely to pose biological risks [44]. The biotechnological industry is a modern industry with biological risks [33].

In South Korea, the tasks, workplaces, and work environments of industries with biological risks have narrow interpretations, but considering that vectors can transport pathogens to a wide range of places or environments, it is necessary to develop comprehensive control measures.

Regarding transportation-affiliated industries, Acke [12] found that flight attendants, drivers, and sailors were exposed to excessive risks, but these industries are not generally recognized as industries with high biological risks. The transportation-affiliated industries were investigated only in 2020, and several transport-related workers had COVID-19 at that time [4]. Even if the workers in transportation-affiliated industries are not exposed to high concentrations of pathogens as in a hospital, jobs with frequent encounters with people may pose a high risk of infection depending on the infectivity of the pathogen, and the workers may be vulnerable to new infectious diseases. As the risk of a new infectious disease epidemic grows, it is necessary to identify jobs involving frequent contact with people in order to develop control measures.

This study may be limited by the structural limitations of industrial accident statistics. First, the industry classifications are not standardized, and the industry names and classifications are not strictly managed. The industry classification system is managed by administrators, not standard experts, and is often revised once a year. In some cases, information can be missing because of changes in the name and code of a specific industry. Recently, there has been a tendency to integrate industry divisions with similar accident rates into one division, making it more difficult to determine the risk of hazards for each individual industry. Second, the classifications of occupational diseases used in industrial accident statistics are neither standardized nor inconsistent. The classifications of occupational diseases are a mixture of classifications based on names of causes, such as mercury and lead, and classifications based on disease names such as occupational cancer and occupational dermatoses. In classifications based on disease names, the impact of biological hazards cannot be measured. Third, the classifications of occupational diseases caused by biological hazards was too broad to be grouped into a single classification. Finally, because industrial accident approval has an impact on the announcement of industrial accidents, there is a possibility of a bias.

The limitation arising from not using raw statistical data cannot be denied. Unlike Chung [8], who was affiliated with the Korea Occupational Safety and Health Agency and was able to use raw data of the industrial accident reports, in this study, only the published data of industrial accident statistics were used. In addition, as the published dataset can be accessed by anyone, it has excellent universality and scalability as research data; however, demographic variables such as sex and age cannot be cross-interpreted. Industrial accident statistics are provided by microdata, but the microdata are published only for a short period of time (2017–2019); in microdata, industry information is announced only up to section, and the detailed classification of disease is different from announced statistics.

In future research, if industries are reclassified using a standard classification, even at the expense of some data loss, the annual trends of change may be clearly observed. In addition, demographic exogenous variables other than the industry as well as other factors influencing infectious diseases must be examined using extended announced data or raw data. 

## 5. Conclusions

In this study, the incidence and fatality rates of disease classifications, including infectious diseases, were calculated by year and industry division using industrial accident statistics, and the relative importance and annual trends of infectious diseases were analyzed. In addition, risk groups were derived by applying the k-means clustering technique based on log values of incidence and fatality rates by industry, and appropriate control measures were discussed for the calculated risk groups.

The infectious disease classification ranked high among occupational disease classifications based on the number of incidences and fatalities. The incidence rate is increasing in the period including the pandemic year, and the fatality rate is continually increasing compared to all occupational diseases. Therefore, among occupational diseases, infectious diseases still require control measures, and it is too early to discuss the decrease in legal control measures.

Risk groups were classified into five groups (groups 1–5): (almost) zero risk, low incidence/low fatality, low incidence/high fatality, high incidence/low fatality, and high incidence/high fatality, respectively. For the high-fatality Group 3, it is necessary to find and improve hazards, considering that this group included industries that are not well known. It is necessary to expand the legal coverage for industries, such as in Groups 4 and 5, that are not legally controlled and are highly susceptible to outbreaks.

This study was based on industrial accident statistics and limited by the structural limitations of industrial accident statistics. In the future, further in-depth research on the impact on risk is needed to further standardize the specificity of the industry and consider temporal characteristics. In addition, a comprehensive review of exogenous variables using raw data is needed.

## Figures and Tables

**Table 1:** Biological hazard control system under the Occupational Safety and Health Act of Korea.

Condition of Legal Management		Basis Clause in Occupational Safety and Health Regulation
Classification	Content	
Task	Medical practice	Article 593 paragraph 1
	Blood test	Article 593 paragraph 2
	Handling of patient’s specimen	Article 593 paragraph 3
	Handling of pathogens for research	Article 593 paragraph 4
	Cattle-breeding	Article 593 paragraph 6 (citing article 592 paragraph 4 sub-paragraph c)
	Slaughtering	Article 593 paragraph 6 (citing article 592 paragraph 4 sub-paragraph c)
Workplace	Group accommodation	Article 593 paragraph 5
	Field	Article 593 paragraph 6 (citing article 592 paragraph 4 sub-paragraph a)
Work environment	Possibility of direct or indirect contact with wild rodents	Article 593 paragraph 6 (citing article 592 paragraph 4 sub-paragraph b)

**Table 2:** Number and rate of illness and fatality by occupational disease type in the industrial accident statistics (2001–2020).

Detailed Classification of Occupational Disease		Number		Rate	
Name	Code of Korean Statistical Information Service	Illness	Fatality	Incidence (per 100,000)	Case Fatality (%)
Total occupational disease	15118AC3BM	44,733	9821	15.26	21.95
Pneumoconiosis	15118AC3BMAA	25,294	8230	8.63	32.54
Noise-induced hearing loss	15118AC3BMAB	11,676	0	3.98	0
Abnormal pressure	15118AC3BMAC	100	2	3.41 × 10^−2^	2.00
Vibration illness	15118AC3BMAD	530	0	1.81 × 10^−1^	0
Other physical factors	15118AC3BMAE	385	58	1.31 × 10^−1^	15.06
Carbon disulfide	15118AC3BMAF	187	97	6.38 × 10^−2^	51.87
Trichloroethylene	15118AC3BMAG	21	8	7.16 × 10^−3^	38.10
Other organic compounds	15118AC3BMAH	148	21	5.05 × 10^−2^	14.19
Benzene	15118AC3BMAI	107	51	3.65 × 10^−2^	47.66
Tar	15118AC3BMAJ	2	1	6.82 × 10^−4^	50.00
Diisocyanates	15118AC3BMAL	90	15	3.07 × 10^−2^	16.67
Asbestos	15118AC3BMAM	591	266	2.02 × 10^−1^	45.01
Other chemicals	15118AC3BMAN	408	46	1.39 × 10^−1^	11.27
Lead	15118AC3BMAO	30	1	1.02 × 10^−2^	3.33
Mercury	15118AC3BMAP	16	0	5.46 × 10^−3^	0
Chrome	15118AC3BMAQ	88	21	3.00 × 10^−2^	3.86
Cadmium	15118AC3BMAR	35	2	1.19 × 10^−2^	5.71
Manganese	15118AC3BMAS	15	6	5.12 × 10^−3^	40.00
Infectious disease	15118AC3BMAT	2905	141	9.91 × 10^−1^	4.85
Toxic hepatitis	15118AC3BMAV01	11	0	3.75 × 10^−3^	0
Occupational dermatoses	15118AC3BMAU	360	0	1.23 × 10^−1^	0
Occupational cancer	15118AC3BMAU00	1376	756	4.69 × 10^−1^	54.95
Other occupational diseases	15118AC3BMAV	358	99	1.22 × 10^−1^	27.65

**Table 3:** Number, rate, and change of rate of illness and fatality of infectious diseases by year according to industrial accident statistics.

Year	Illness	Fatality	Number of Workers	Incidence Rate (per 100,000)	Case Fatality Rate (%)	Change in Incidence Rate (%)	Change in Case Fatality Rate (%)
2001	108	6	10,581,186	1.02	5.56	-	-
2002	93	7	10,571,279	8.80 × 10^−1^	7.53	−13.81	35.48
2003	132	5	10,599,345	1.25	3.79	41.56	−49.68
2004	167	2	10,473,090	1.59	1.20	28.04	−68.38
2005	100	1	11,059,193	9.04 × 10^−1^	1.00	−43.29	−16.50
2006	131	6	11,688,797	1.12	4.58	23.94	358.02
2007	188	7	12,528,879	1.50	3.72	33.89	−18.71
2008	134	5	13,489,986	9.93 × 10^−1^	3.73	−33.80	0.21
2009	427	8	13,884,927	3.08	1.87	209.59	−49.79
2010	228	17	14,198,748	1.61	7.46	−47.78	297.97
2011	168	11	14,362,372	1.17	6.55	−27.16	−12.18
2012	133	8	15,548,423	8.55 × 10^−1^	6.02	−26.87	−8.13
2013	134	8	15,449,228	8.67 × 10^−1^	5.97	1.40	−0.75
2014	107	14	17,062,308	6.27 × 10^−1^	13.08	−27.70	119.16
2015	77	3	17,968,931	4.29 × 10^−1^	3.90	−31.67	−70.22
2016	80	7	18,431,716	4.34 × 10^−1^	8.75	1.29	124.58
2017	95	4	18,560,142	5.12 × 10^−1^	4.21	17.93	−51.88
2018	79	7	19,073,438	4.14 × 10^−1^	8.86	−19.08	110.44
2019	84	9	18,725,160	4.49 × 10^−1^	10.71	8.31	20.92
2020	240	6	18,974,513	1.26	2.50	181.96	−76.67

**Table 4:** Number, rate and change of rate of illness and fatality of whole occupational diseases and ratio of infectious diseases to whole occupational diseases by year.

Year	Illness	Fatality	Number of Workers	Incidence Rate (per 100,000)	Case Fatality Rate (%)	Incidence Ratio of Infectious Diseases to All Occupational Diseases (%)	Fatal Ratio of Infectious Diseases to All Occupational Diseases (%)
2001	1542	415	10,581,186	14.57	26.91	7.00	1.45
2002	1351	407	10,571,279	12.78	30.13	6.88	1.72
2003	1905	482	10,599,345	17.97	25.30	6.93	1.04
2004	2492	446	10,473,090	23.79	17.90	6.70	0.45
2005	2524	455	11,059,193	22.82	18.03	3.96	0.22
2006	2174	524	11,688,797	18.60	24.10	6.03	1.15
2007	2098	480	12,528,879	16.75	22.88	8.96	1.46
2008	1653	463	13,489,986	12.25	28.01	8.11	1.08
2009	1746	431	13,884,927	12.57	24.68	24.46	1.86
2010	1576	447	14,198,748	11.10	28.36	14.47	3.80
2011	1592	430	14,362,372	11.10	27.01	10.55	2.56
2012	1500	410	15,548,423	9.65	27.33	8.87	1.95
2013	1414	466	15,449,228	9.15	32.96	9.48	1.72
2014	1732	507	17,062,308	10.15	29.27	6.18	2.76
2015	1959	514	17,968,931	10.90	26.24	3.93	0.58
2016	2234	478	18,431,716	12.12	21.40	3.58	1.46
2017	3054	582	18,560,142	16.45	19.06	3.11	0.69
2018	3368	628	19,073,438	17.66	18.65	2.35	1.11
2019	4035	607	18,725,160	21.55	15.04	2.08	1.48
2020	4784	649	18,974,513	25.21	13.57	5.02	0.92

**Table 5:** Characteristics of risk groups by incidence and fatality rate of infectious diseases in Industrial Accident Statistics.

Group	Character	Incidence Rate (per 100,000)			Case Fatality Rate (%)		
		Min.	Max.	Mean	Min.	Max.	Mean
1	(Almost) Risk zero	0	5.46 × 10^−2^	1.82 × 10^−3^	0	0	0
2	Low incidence/low fatality	9.65 × 10^−2^	6.10 × 10^−1^	2.39 × 10^−1^	0	0	0
3	Low incidence/high fatality	4.58 × 10^−2^	5.06 × 10^−1^	2.52 × 10^−1^	5.00	100	40.28
4	High incidence/low fatality	1.07	23.57	6.49	0	3.92	1.61
5	High incidence/high fatality	6.56 × 10^−1^	8.63	2.54	7.69	100	35.02

**Table 6:** Industry division by risk group and the incidence and fatality rates by industry division.

Group	Industry Division (Code Identifier of Korean Statistical Information Service, Survey Period)	Incidence Rate (per 100,000)	Case Fatality Rate (%)
1	Mining of limestone, metals, non-metals and other mining (AAG, 2017–2020)	0	0
	Mining of metals and non-metals (AAB, 2001–2016)	0	0
	Quarrying (AAC, 2001–2016)	0	0
	Mining of limestone (AAD, 2001–2016)	0	0
	Tobacco manufacturing (BAn, 2001–2017)	0	0
	Wood and paper products manufacturing (BAC000, 2019–2020)	0	0
	Pulp and paper manufacturing and bookbinding and printed matter processing industry (BAp, 2001–2017)	0	0
	Pulp and paper manufacturing industry (BAp0, 2018–2018)	0	0
	Publishing, printing, bookbinding and print processing industry (BAD0, 2018–2020)	0	0
	Printing (BAE, 2001–2011)	0	0
	Chemical and rubber product manufacturing (BAF0, 2019–2020)	0	0
	Pharmaceuticals, cosmetics, briquettes and petroleum products manufacturing (BAH00, 2020–2020)	0	0
	Coke and coal gas manufacturing industry (BAJ, 2001–2011)	0	0
	Glass, porcelain and cement manufacturing (BAq0, 2019–2019)	0	0
	Ceramics, other ceramic products and cement manufacturing (BAK0, 2018–2018)	0	0
	Cement manufacturing (BAM, 2001–2017)	0	0
	Electric machine equipment, electronic products, meters, optical machinery, and other precision equipment manufacturing (BAQ0, 2020–2020)	5.46 × 10^−2^	0
	Coke, briquettes and petroleum refineries manufacturing (BAZ00, 2012–2019)	0	0
	Briquette and coagulated solid fuel manufacturing (BAX, 2001–2011)	0	0
	Electricity, gas and water business (CAA, 2001–2011)	0	0
	Electricity, gas, steam and water business (CAA00, 2012–2020)	0	0
	Automobile transport, courier and quick service business (EAN, 2017–2018)	0	0
	Air transportation business (EAH, 2001–2017)	0	0
	Warehouse and transportation related service business (EAI0, 2019–2019)	0	0
	Small cargo transport, courier and quick service business (EAM, 2001–2016)	0	0
	Fishery, aquaculture and fishery related services (GAC, 2017–2018)	0	0
	Fishing (GAD, 2019–2020)	0	0
	Fishing (GAA, 2001–2016)	0	0
	Consignment sales of agricultural and marine products (JAP, dummy code)	0	0
	United States Forces Korea (JAJ, 2001–2020)	0	0
2	Textile or textile product manufacturing business (A) (BAo, 2001–2018)	1.49 × 10^−1^	0
	Textile or textile product manufacturing business (BAoo, 2019–2020)	2.83 × 10^−1^	0
	Newspaper/money issuance, publishing business and printing business (BAD, 2001–2017)	1.85 × 10^−1^	0
	Pharmaceuticals, cosmetics, fragrances and tobacco manufacturing (BAH0, 2018–2019)	6.10 × 10^−1^	0
	Rubber product manufacturing (BAG, 2001–2018)	9.65 × 10^−2^	0
	Glass manufacturing (BAq, 2001–2018)	1.93 × 10^−1^	0
	Ceramics and other ceramic products manufacturing (BAK, 2001–2017)	1.36 × 10^−1^	0
	Machine tools, non-metallic mineral products, metal products manufacturing and metal processing (BAr0, 2018–2018)	1.06 × 10^−1^	0
	Machine tools, non-metallic minerals and metal products manufacturing (BAr00, 2019–2019)	2.05 × 10^−1^	0
	Machine tools, metal and non-metallic minerals products manufacturing (BAr000, 2020–2020)	4.64 × 10^−1^	0
	Plating (BAO, 2001–2018)	1.34 × 10^−1^	0
	Electric machine equipment, precision equipment, and electronic products manufacturing (BAQ00, 2020–2020)	1.09 × 10^−1^	0
	Transportation machinery and equipment manufacturing, automobile and motorcycle repairing (BAT0, 2018–2018)	2.02 × 10^−1^	0
	Handicraft manufacturing (BAV, 2001–2018)	3.57 × 10^−1^	0
	Handicraft and other products manufacturing (BAV0, 2019–2020)	3.66 × 10^−1^	0
	Automobile and motorcycle repairing (BAZ, 2001–2017)	1.42 × 10^−1^	0
	Railroad, track and ropeway transportation business (EAA, 2001–2017)	2.37 × 10^−1^	0
	Railroad, track, ropeway and air transportation business (EAA0, 2018–2019)	4.13 × 10^−1^	0
	Land and water transport business (EAN0, 2019–2020)	1.40 × 10^−1^	0
	Water transport, port unloading and cargo handling business (EAF, 2001–2018)	1.63 × 10^−1^	0
	Warehousing business (EAJ, 2001–2018)	1.78 × 10^−1^	0
	Education service business (JAG. 2001–2019)	3.94 × 10^−1^	0
3	Wood products manufacturing (BAC00, 2012–2018)	2.47 × 10^−1^	100.00
	Chemical manufacturing (BAF, 2001–2018)	2.81 × 10^−1^	25.00
	Pharmaceutical and cosmetic fragrance manufacturing (BAH, 2001–2017)	2.39 × 10^−1^	100.00
	Non-metallic mineral products and metal products manufacturing and metal processing industry (BAr, 2001–2017)	4.43 × 10^−1^	22.22
	Metal smelting (BAL, 2001–2020)	1.45 × 10^−1^	100.00
	Metal material manufacturing (BAN, 2001–2016)	2.10 × 10^−1^	50.00
	Machine tool manufacturing (BAP, 2001–2017)	2.94 × 10^−1^	5.00
	Electrical machinery manufacturing (BAQ, 2001–2017)	1.72 × 10^−1^	20.00
	Electronics manufacturing (BAR, 2001–2017)	4.58 × 10^−2^	75.00
	Shipbuilding and repairing (BAS, 2001–2020)	2.20 × 10^−1^	57.14
	Transportation machinery and equipment manufacturing (BAT, 2001–2017)	3.54 × 10^−1^	9.52
	Textile or textile product manufacturing (B) (BAY, 2001–2018)	1.51 × 10^−1^	33.33
	Other manufacturing (BAs, 2001–2018)	1.53 × 10^−1^	66.67
	Construction industry (DAB, 2001–2020)	3.23 × 10^−1^	7.18
	Passenger car transport business (EAB, 2001–2016)	1.76 × 10^−1^	25.00
	Freight car transportation business (EAC, 2001–2016)	3.17 × 10^−1^	100.00
	Transportation-related service business (EAI, 2001–2018)	3.75 × 10^−1^	25.00
	Telecommunications business (EAK, 2001–2020)	4.97 × 10^−1^	25.00
	Finance and insurance (KAA, 2001–2020)	9.52 × 10^−2^	27.27
	Professional technical service business (JAE, 2001–2019)	1.74 × 10^−1^	15.79
	Wholesale, retail and consumer goods repairing business (JAH, 2001-2019)	1.47 × 10^−1^	20.51
	Wholesale, retail, food and lodging business (JAH0, 2020–2020)	4.54 × 10^−1^	6.67
	Real estate business and rental business (JAI, 2001–2020)	1.16 × 10^−1^	50.00
	Business service (CAA03, 2018–2019)	1.55 × 10^−1^	33.33
	Various other business (JAD, 2001–2020)	5.06 × 10^−1^	7.34
4	Wood product manufacturing (BAC, 2001–2011)	3.42	0
	Railroad, air transportation, warehousing and transportation-related service business (EAA00, 2020–2020)	2.90	0
	Forestry (FAA, 2001–2020)	23.57	2.06
	Aquaculture and fishery related services (GAB, 2001–2016)	1.80	0
	Agriculture (HAA, 2001–2020)	10.81	1.83
	Comprehensive management of buildings, etc. business (JAA, 2001–2018)	1.07	3.74
	Facility management business and business service (JAA00, 2020–2020)	1.57	0
	Sanitation and similar service business (JAB, 2001–2018)	13.90	3.24
	Professional technical, health, education, recreation service business (JAE0, 2020–2020)	3.38	1.63
	Health and social welfare business (JAF, 2001–2019)	4.64	1.23
	Business of the state and local governments (CAA02, 2012–2020)	4.36	3.92
5	Coal mining and quarrying (AAF, 2017–2020)	8.63	100.00
	Coal mining (AAA, 2001–2016)	1.29	100.00
	Other mining (AAE, 2001–2016)	3.78	25.00
	Food manufacturing (BAA, 2001–2020)	6.97 × 10^−1^	12.50
	Sawmill and veneer manufacturing (BAB, 2001–2011)	4.32	25.00
	Measuring instruments, optical instruments, and other precision instruments manufacturing (BAU, 2001–2017)	6.70 × 10^−1^	22.22
	Comprehensive building management, sanitation and similar service business (JAA0, 2019–2019)	8.39 × 10^−1^	12.50
	Golf course and racetrack operation business (JAC, 2001–2011)	2.27	16.67
	Overseas dispatcher (JAL, 2001–2020)	2.26	28.57
	Entertainment, culture and sports related business (CAA01, 2012–2019)	6.56 × 10^−^^1^	7.69

## Data Availability

Not applicable.
